# The Use of Patient-Facing Teleconsultations in the National Health Service: Scoping Review

**DOI:** 10.2196/15380

**Published:** 2020-03-16

**Authors:** Micheal O'Cathail, M Ananth Sivanandan, Claire Diver, Poulam Patel, Judith Christian

**Affiliations:** 1 School of Medicine University of Nottingham Nottingham United Kingdom; 2 Department of Oncology & Radiotherapy Nottingham University Hospitals NHS Trust Nottingham United Kingdom; 3 School of Medicine & Health Sciences University of Nottingham Nottingham United Kingdom

**Keywords:** telehealth, telemedicine, teleconsultation, scoping review

## Abstract

**Background:**

The National Health Service (NHS) *Long-Term Plan* has set out a vision of enabling patients to access digital interactions with health care professionals within 5 years, including by video link.

**Objective:**

This review aimed to examine the extent and nature of the use of patient-facing teleconsultations within a health care setting in the United Kingdom and what outcome measures have been assessed.

**Methods:**

We conducted a systematic scoping review of teleconsultation studies following the Joanna Briggs Institute methodology. PubMed, Scopus, the Cochrane Library, and the Cumulative Index to Nursing and Allied Health Literature were searched up to the end of December 2018 for publications that reported on the use of patient-facing teleconsultations in a UK health care setting.

**Results:**

The search retrieved 3132 publications, of which 101 were included for a full review. Overall, the studies were heterogeneous in design, in the specialty assessed, and reported outcome measures. The technology used for teleconsultations changed over time with earlier studies employing bespoke, often expensive, solutions. Two-thirds of the studies, conducted between 1995 and 2005, used this method. Later studies transitioned to Web-based commercial solutions such as Skype. There were five outcome measures that were assessed: (1) technical feasibility, (2) user satisfaction, (3) clinical effectiveness, (4) cost, (5) logistical and operational considerations. Due to the changing nature of technology over time, there were differing technical issues across the studies. Generally, teleconsultations were acceptable to patients, but this was less consistent among health care professionals. However, among both groups, face-to-face consultations were still seen as the gold standard. A wide range of clinical scenarios found teleconsultations to be clinically useful but potentially limited to more straightforward clinical interactions. Due to the wide array of study types and changes in technology over time, it is difficult to draw definitive conclusions on the cost involved. However, cost savings for health care providers have been demonstrated by the goal-directed implementation of teleconsultations. The integration of technology into routine practice represents a complex problem with barriers identified in funding and hospital reimbursement, information technologies infrastructure, and integration into clinicians’ workflow.

**Conclusions:**

Teleconsultations appear to be safe and effective in the correct clinical situations. Where offered, it is likely that patients will be keen to engage, although teleconsultations should only be offered as an option to support traditional care models rather than replace them outright. Health care staff should be encouraged and supported in using teleconsultations to diversify their practice. Health care organizations need to consider developing a digital technology strategy and implementation groups to assist health care staff to integrate digitally enabled care into routine practice. The introduction of new technologies should be assessed after a set period with service evaluations, including feedback from key stakeholders.

## Introduction

Telemedicine is a branch of medicine, which concerns the use of information technologies (IT) in all aspects of medical care and education. A literary consensus defined telemedicine as: “…a subset of telehealth, uses communications networks for delivery of health care services and medical education from one geographical location to another, primarily to address challenges such as uneven distribution and shortage of infrastructural and human resources” [1]. Common examples of telemedicine include using telephones for patient interaction, videoconferencing with multidisciplinary team meetings, and the use of email in professional practice. Many of these technologies are considered integral to routine clinical practice. The National Health Service (NHS) refers to telemedicine as being synonymous with teleconsultations, involving a video link with patients [2]. To avoid confusion with other definitions, this review shall use the term teleconsultations rather than telemedicine.

Teleconsultations have the potential to improve access to medical care and reduce travel and costs for patients while maintaining the quality of care [3]. The NHS’s recently published *Long-Term Plan* has set out a vision of how to transform outpatient care using technology. It states the desire to offer all patients the choice of digital interaction, including the use of teleconsultations, within 5 years, and to remove 30 million face-to-face appointments [4]. With such an ambitious plan, this review looks at the UK evidence of teleconsultation use for patient-facing interactions.

Although a systematic review may provide evidence for how effective an intervention is based on a predetermined study type, usually a randomized controlled trial (RCT), a scoping review can answer the broader question of what is already known; what the extent, nature, and range of intervention use is, and allows for greater inclusivity of different study types [5]. The objective of this review was to map the available evidence in relation to the use of patient-facing teleconsultations in the NHS. A review of the literature before commencing this review identified no existing systematic or scoping review that addressed this issue.

## Methods

### Methodological Framework

This review was guided by the methodological framework devised by Arksey and O’Malley [5], and further amendments that were contributed by Levac et al [6] and the Joanna Briggs Institute on conducting systematic scoping reviews [7]. This framework consists of a number of consecutive stages: (1) identifying the research question, (2) identifying relevant studies; (3) study selection; (4) charting the data; (5) collating, summarizing, and reporting results. This methodology summarizes the evidence available on a topic to convey the breadth and depth of that topic. We used the Preferred Reporting Items for Systematic Reviews and Meta Analyses (PRISMA) extension for Scoping Reviews checklist to report our results [8]. At present, the international Prospective Register of Systematic Reviews does not publish protocols for scoping reviews.

### Identifying the Research Question

The purpose of this review was to find out what health care settings in the United Kingdom teleconsultations have been used in. The broad research questions of this review were as follows: What is the extent and nature of use of patient-facing teleconsultations within a health care setting in the United Kingdom and what outcome measures have been assessed?

### Identifying the Relevant Studies

#### Information Sources and Search Strategies

As this review is interested only in the UK-based experience of teleconsultations, the study’s search strategy was restricted to the United Kingdom or NHS affiliated authors. Databases searched were PubMed, Scopus, Cochrane library, and the Cumulative Index of Nursing and Allied Health Literature. Studies up to the end of 2018 were included with no predetermined lower range. The search strategy was developed in PubMed and translated into other databases. This is outlined in Table 1. Search results were exported to the Mendeley reference manager (Elsevier) and duplicated results were removed.

**Table 1:** PubMed search strategy.

Search field	Search term
Intervention	(Teleconsultation* OR telemedicine OR virtual clinic* OR video clinic* OR virtual consultation* OR video consultation*)
Restricted to UK-based authors	AND (UK[Affiliation] OR NHS[Affiliation] OR United Kingdom[Affiliation])
Date restrictions	Studies up to December 31, 2018
Total articles	2065

### Eligibility Criteria

#### Types of Participants

This review included all participants that used teleconsultations in a health care setting. The only restriction was geography, as the area of the interest is specifically the NHS in the United Kingdom. All studies which used teleconsultations for direct patient-facing care were included. Any studies that used teleconsultations in a nonpatient facing capacity (eg, professional to professional teleconsultations for multidisciplinary meetings) were excluded.

This review was limited to studies that were conducted in the United Kingdom, and there was no restriction on the specialty or type of professionals involved in the consultations. Studies up to the end of 2018 were eligible for inclusion. All studies up to December 31, 2018, were included, and the date of the last search was on February 7, 2019. Study titles and abstracts were independently screened by two reviewers (MOC and MAS) based on predetermined inclusion and exclusion criteria, which are outlined in Table 2. Where abstracts were not available, these articles were excluded.

**Table 2:** Inclusion and exclusion criteria.

Search Parameter	Inclusion criteria	Exclusion criteria
Population	Any health care setting	Non–health care setting
Date	Up to December 31, 2018	N/A^a^
Study type	No restrictions	N/A
Intervention	Teleconsultations involving real-time video link with patients	No video link–based telemedical intervention Not patient-facing (eg, teleconference multidisciplinary team meeting) Not real time (eg, store and forward models in teledermatology)
Location	United Kingdom/NHS^b^	Non-UK-based studies

^a^N/A: not applicable.

^b^NHS: National Health Service.

#### Study Type

There was no restriction on the study type eligible for inclusion.

#### Study Selection

All studies up to December 31, 2018, were included, and the date of the last search was on February 7, 2019. Study titles and abstracts were independently screened by two reviewers (MC and MS) based on predetermined inclusion and exclusion criteria, which are outlined in Table 2. Where abstracts were not available, these articles were excluded. If the study suitability was not clear from the abstract, the full paper was requested for review. Disagreements between reviewers were resolved through consensus. The reasons for exclusion were only recorded at the full-text stage.

### Charting the Data

The research team developed a data extraction tool that included the following items: (1) article identifiers (ie, year of publication, author, and title), (2) study identifiers (ie, study design and sample size), (3) setting/population (ie, area of medical specialty), (4) outcome measures assessed, and (5) brief article synopsis.

Data were extracted by one reviewer and verified by a second. The table charting of these articles in temporal order is shown in Multimedia Appendix 1 [9-109].

### Collating, Summarizing, and Reporting the Results

A descriptive numerical summary of the characteristics of the included studies was performed. Tables and graphs were created to reflect the overall number of studies included, study designs and settings, publication years, and the outcomes reported. In line with the methodology of scoping reviews, an assessment of the quality of the included studies was not performed.

### Statement of Patient and Public Involvement

This research was conducted without patient involvement. Patients were not invited to comment on the study design and were not consulted to develop patient-relevant outcomes or interpret the results. Patients were not invited to contribute to the writing or editing of this document for readability or accuracy.

## Results

### Study Characteristics

**Figure 1:**
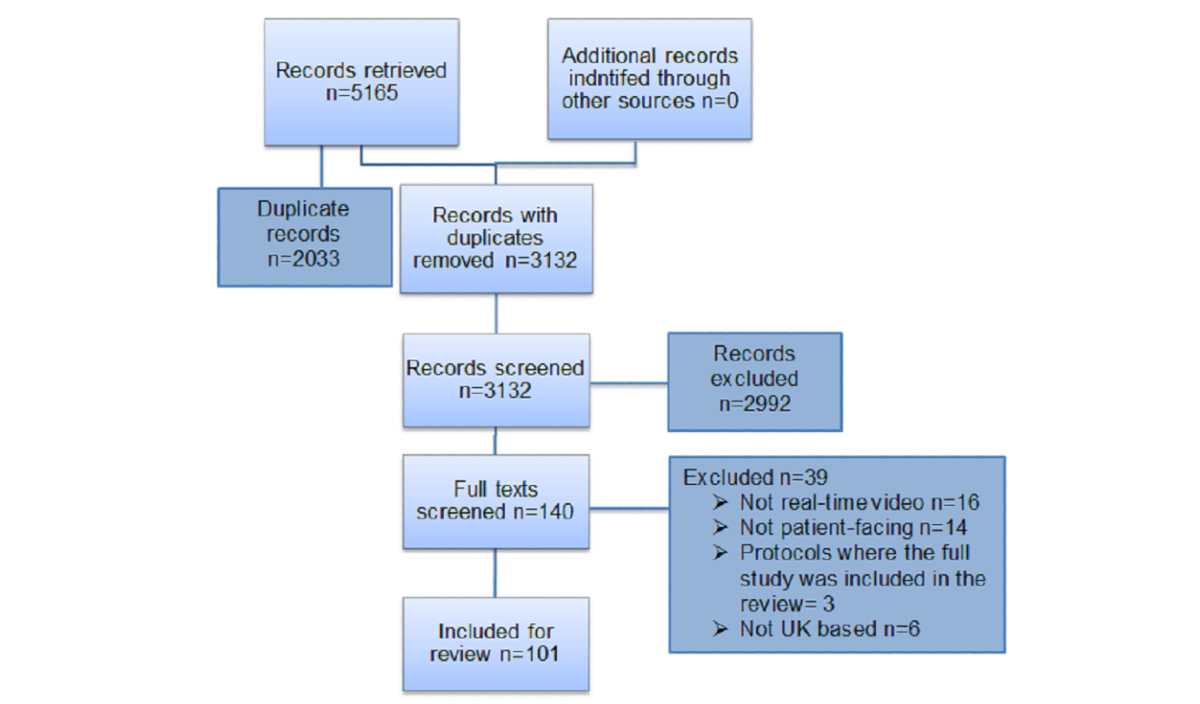
The Preferred Reporting Items for Systematic Reviews and Meta-Analyses flow chart—teleconsultations in the National Health Service.

A total of 3132 articles were retrieved. In total, 140 full texts were retrieved, with 101 meeting the inclusion criteria for review. The PRISMA flowchart is shown in Figure 1.

### What is the Extent and Nature of the Use of Patient-Facing Teleconsultations Within a Health Care Setting in the United Kingdom?

There were a total of 101 studies across 24 different specialties included in the review starting in 1995 and ending in 2018. There was a large variation in study type, shown in Table 3. Pilots, audits, service reports, and case series/reports represented more than half of the articles included for review, whereas there were 13 RCTs [21,25,26,40,41,59-61,64,71,74,84,87]. Of these RCTs, there were 8 papers written about 4 RCTs [25,26,40,41,59,60,64,71]; therefore, only 9 could be considered unique study populations. Of these, 4 [61,74,84,87] had 30 or fewer participants, including one with 6 [61] and another with 11 participants [87].

Accident and emergency (A&E) was the most common single specialty studied with 19 articles, followed by psychiatry/psychology, neurology, and acute stroke. There were 15 studies that included more than one specialty, sometimes with a mix of primary and secondary care. Most specialties, however, were represented in 2 or fewer papers. These are shown in Table 4.

The timeline of published articles shows that there was significant interest in the potential of teleconsultations in the early 2000s, but this reduced significantly from 2003 until 2017 when publication numbers began to rise again. This is shown in Table 5.

**Table 3:** Summary of article types.

Article type	Count, n
Systematic review	4
Reviews	7
Randomized control trial	13
Cohort	2
Single-cluster, balanced crossover, blind	1
Mixed method	2
Qualitative study	12
Case-control	8
Retrospective study	5
Service report	2
Audit	3
Pilot	30
Case report/series	6
Descriptive study	3
Study protocol	3
Total	101

**Table 4:** Publications by specialty.

Specialty	Publications, n	Participants, n
Accident and emergency	19	7394
Multiple	15	2832
Psychiatry/psychology	15	264
Neurology	8	471
Stroke	7	356
General practice	6	884
Pediatrics	5	662
Dermatology	4	678
Orthopedics	2	71
Nephrology	2	16
Respiratory	2	71
Rehabilitation	2	1
Speech and language therapy	2	15
Ophthalmology	2	47
Rheumatology	2	120
Gastrointestinal/hepatology	1	80
Endocrinology	1	480
Care of the elderly	1	Unspecified
Genetics	1	37
Dietetics	1	30
Dentistry	1	25
Transplant medicine	1	180
Oncology	1	8

**Table 5:** Number of publications by year

Year	Publications, n
1995-2001	39
2002-2007	32
2008-2013	15
2014-2018	15
Total	101

### What Outcome Measures Have Been Assessed in UK-Based Studies?

There are five main outcomes that were identified. These are (1) technical feasibility; (2) user acceptability; (3) clinical effectiveness; (4) economic assessment; and (5) logistical and operational considerations. Each section will provide an overview of the article types and a narrative summary of the findings of these studies.

#### Technical Feasibility

Technical feasibility relates to practical issues with using the technology used for teleconsultations, for example, where a study reports difficulties with the audiovisual link. We identified 18 articles, which reported aspects related to technical feasibility of which there were 8 pilots [14,32,37,39,43,56,82,86], 2 case reports/series [17,36], 1 descriptive study [46], 1 RCT [61], 2 systematic reviews [73,75], 2 nonsystematic reviews [54,67], 1 mixed method study [105], and 1 study protocol [38].

In total, 11 studies, including both systematic reviews, reported acceptable quality and reliability of the teleconsultation link. In total, 3 studies reported problems with audio or video quality, and this tended to follow the perceived importance of that deficiency. For instance, one study reported that the image quality of a video link was so poor such that 46% of dermatologists felt the diagnostic accuracy would be adversely affected [14]. In another, among peritoneal dialysis patients, poor image quality affected its utility in assessing Tenckhoff catheter sites [61]. A teletherapy study found that sound lag affected the flow of therapy at times, although it concluded that this was not prohibitive to continuing the session [43].

In a study designed to assess the feasibility of a dedicated teleconsultation link in a police college, the authors reported significant reliability issues, where only half of the intended patients were able to be seen by teleconsultation due to unspecified technological failures [86]. A more recent study, using commercial Web-based video calling technology, found that technical issues were minor but often prohibitive to proceeding with the consultation if not rectified. Workarounds by the clinician rectified these; loss of sound on two occasions was overcome by using a telephone for audio, and lack of video was found to be due to the patient forgetting to turn on the Web camera [105].

#### User Satisfaction and Experience

Satisfaction and user experience with teleconsultations was reported in 43 articles of which there were 17 pilots [9,14-16,23,39,43,45,50,52,56,70,77,81,85,97,108], 11 qualitative studies [33,51,57,76,89,93,98,100,101,107,109], 4 RCTs [21,40,59,61], 2 mixed method studies [102,105], 3 trial protocols [38,68,106], 2 case-control studies [42,69], 1 cohort [18], 1 systematic review [31], 1 nonsystematic review [54], and 1 descriptive study [47].

Satisfaction was assessed using feedback questionnaires in 23 articles, including 3 RCTs. Of these, 19 reported high levels of satisfaction with the medium. In 2 RCTs, satisfaction in the teleconsultation arm was actually greater than the face-to-face group [21,59], whereas another found no difference [40]. By contrast, only 1 small RCT found that patients were less satisfied with teleconsultations due to poor image and audio quality [61]. One pilot found that, while patients were satisfied, health care staff were uncomfortable with it; citing that they felt more *on show* to senior colleagues and families than would be normal in a face-to-face appointment [70].

A systematic review of patient satisfaction with teleconsultations concluded that although the published evidence suggests that teleconsultations appear to have high satisfaction rates in a variety of settings, we should be cautious about interpreting that as a true reflection of real life [31]. The authors suggest that most studies conducted tried to minimize the inconvenience for those taking part, and often, patients were seen both in person and by teleconsultation.

Patients and staff may be satisfied with teleconsultations, but that is not to say that they are preferable to face-to-face consultations. Several studies found that patients were satisfied with teleconsultations but also that they would still want the option to attend in person as they believe it to be the *gold standard* [77,107,109].

Qualitative studies exploring users’ experiences of teleconsultations find that the main benefits commonly reported by patients are convenience, reduced travel, and greater accessibility to specialist care and improved flexibility of appointments, allowing minimal disruption to daily life [102,107]. Several studies found that the medium allowed patients to *open up* more than face-to-face consultations and that they felt empowered to ask more questions [57,107]. Among staff, a greater sense of job satisfaction and a reduced burden of travel have been reported [101]. Among a cancer population, participants reported a preference for receiving *bad news* in the comfort of their homes rather than in hospital [105].

By contrast, among a teenaged population being treated for chronic fatigue, participants raised concerns about privacy, fearing that they might be overheard by family. Their parents worried that the connection might not be secure enough to ensure privacy, while some health professionals thought it was an invasion of patients’ personal space [107]. There was the awareness that teleconsultations had certain *physical* limitations; the qualitative analysis from the large RCT by Wallace et al [59] found, either due to patient expectation or physician need, that the inability to perform physical examinations limited its usefulness [76]. A recent study found that physicians often restricted who was offered teleconsultations based on preconceived impracticalities, or they simply refused to participate in them [105].

One study, in which teleconsultations between patients and hospital specialists were facilitated by general practitioners (GPs), concluded that teleconsultations had a different dialogue flow than traditional face-to-face appointments. In particular, the opening phase of the consultation was found to be unfamiliar, leading to interruptions and apologies on both sides while a dialogue flow was established [89]. Morris et al [102] reported that patients and staff could find the medium awkward and uncomfortable when there was no previous relationship built up. The authors concluded that when there were staff changes in service or new-patient appointments, teleconsultations would not be appropriate. Haig-Ferguson et al [107] found that some participants felt teleconsultations were less personal and that the therapist was less *real* over a video link, with the screen acting as a physical and emotional barrier. Paradoxically, the same study found that being physically removed from the therapist allowed other participants to open up more easily. Due to these potential social difficulties, authors have suggested that teleconsultations are more appropriate for follow up appointments [102,107].

A telestroke study found that the utility of teleconsultations in facilitating timely care was acknowledged by families of acute stroke sufferers but that the clinical expertise of the on-site team was important for them to have confidence in the process [98].

#### Clinical Effectiveness

Where articles commented on the efficacy, safety or other clinical outcomes, these were categorized as assessing the clinical effectiveness of teleconsultations in delivering health care. There were 48 articles of which there were 12 pilots [14,15,30,34,43,48,62,63,65,86,88,108], 5 case reports/series [19,22,36,72,99], 7 case-controls [28,42,44,49,53,69,78], 1 qualitative study [10], 2 mixed method [102,105], 4 retrospective studies [35,58,80,92], 3 audits [83,94,95], 7 RCTs [26,40,59,64,74,84,87], 1 single-clustered, blinded crossover design study [79], 1 cohort [66], 2 systematic reviews [73,75], 1 nonsystematic review [54], 1 descriptive study [90], and 1 service report [96].

Within psychiatry, a single-cluster balanced crossover, blind study (where each patient had both a face-to-face and teleconsultation with a different researcher and each researcher was blind to the psychiatric assessment of the other) concluded that there was significant intermethod concordance, confirming its accuracy in psychiatric assessment [79]. This confirms the findings of an earlier systematic review [75].

Teleconsultations in acute stroke management networks are now widespread in the United Kingdom. In total, 3 retrospective studies of a combined 287 patients conducted in the United Kingdom confirm that its implementation has been safe; *door-to-needle time*, morbidity, mortality, and discharge rates were comparable to national standards for acute stroke management [92,94,95]. A novel study exploiting an inherent advantage of teleconsultations describes an international telestroke service between Scotland and New Zealand. In this small case series, there were no negative patient outcomes, and the authors suggest that utilization of the time difference would avoid doctor fatigue [99].

In total, 2 case-control studies in neurology assessed the concordance of diagnosis in both an inpatient and outpatient setting and found 96%-100% of cases were accurately diagnosed and managed via teleconsultation [28,29]. An RCT in a neurology outpatient setting compared face-to-face consultations with teleconsultations and found that the teleconsultation arm generated more investigations despite no difference in the diagnostic category of the cases seen. The authors conclude that this reflected a lack of confidence in their teleconsultation diagnosis [40]. A cohort study of 111 inpatients assessed by video link found no difference in 3-month mortality compared with all other hospital admissions during that time. On follow-up, no patient had their diagnosis or management changed when seen face-to-face, and no difference was seen in the use of hospital services in the following 3 months after discharge [66].

A large multispecialty RCT, by Wallace et al [59], enrolled over 2000 patients. They measured the number of investigations per patient and follow-up rates and, in contrast to the previously mentioned RCT, found that teleconsultations actually resulted in fewer investigations, at a rate of 0.79 per patient. However, this figure is offset by a higher rate of subsequent follow up seen in this group.

In an A&E setting, an RCT found no significant differences in diagnostic accuracy or management when teleconsultations were compared with the traditional model of care [64]. In minor injury units, the use of teleconsultations, connecting with a regional A&E center, allowed the majority of patients to be managed locally, with continued improvements seen with increasing technological familiarity [48,58,62,65].

In rheumatology, 2 studies reported conflicting findings. Graham et al [30] found rheumatologists—using a junior doctor as a proxy—were only 40% accurate in assessments via teleconsultation with physicians missing subtle but clinically important signs of inflammation. A year later, Leggett et al [42] concluded that teleconsultations—using a 3-way consultation between the patient, GP, and specialist—were 97% accurate in diagnosing fibromyalgia, degenerative arthritis, rheumatoid arthritis, and soft tissue disease.

In ophthalmology, some eye conditions such as simple ptosis and strabismus could be accurately assessed in up to 97% of cases. However, more complex eye conditions such as socket problems in patients who had a previous enucleation or those with nonspecific ocular pain were better assessed in a face-to-face consultation [44,49].

In Airedale NHS trust, providing a teleconsultation link between care homes and hospitals reduced nonelective admissions by 1731—a 37% reduction—compared with the same period before the intervention [96]. In a hospital diabetic clinic setting, over 4 years, appointment *did not attend* (DNA) rates were lower (13% vs 28%) in patients choosing to attend by teleconsultation with improved hemoglobin A_1c_ control [102]. In a prison inmate setting, the use of teleconsultations, coupled with other interventions, improved clinical outcomes for those being screened and treated for hepatitis C compared with controls [108].

Real-world data on teleconsultation appointments as a proportion of clinical activity has been rarely reported and ranges from 2% among a diabetic cohort to 22% among postoperative hepatobiliary cancer patients [105].

#### Cost

In total, 19 articles looked at health care provider cost, patient cost, or costs incurred by both. This comprised 5 RCTs [25,26,41,60,71], 3 systematic reviews [73,75,104], 2 nonsystematic reviews [54,91], 1 retrospective study [27], 1 case-control study [78], 1 service report [96], 3 pilots [12,43,55], and 3 protocols [38,68,106]. Nearly all of the studies reported higher costs for health care providers, including all RCTs. The issue of cost is closely related to the technology used, which has changed greatly over the period of this review—from expensive audiovisual systems to the use of smartphones and computers.

Early studies found that the initial cost of suitable videoconferencing equipment was prohibitively expensive. One early study quotes a figure of £48,000 (US $61,439) to establish a teleconsultation link, including videoconferencing unit and integrated services digital network connection charges [26]. Loane et al published the results of an RCT in 2 papers [25,26]. They found that real-time teleconsultations were 5 times more expensive to run for health providers than *store-and-forward* teleconsultation models; £132.10 (US $169) vs £29.60 (US $37.90). Although patients saved time and money due to reduced traveling, health care-associated costs were higher in the real-time arm as they took up more physician time than the store and forward model.

Direct comparison with face-to-face appointments in an outpatient setting has found teleconsultations to be more expensive for health care providers. In total, 3 RCTs and 1 case-control study set in pediatrics, neurology, secondary care outpatients, and A&E concluded that teleconsultations were between 15% and 100% more costly to run [41,60,71,78]. A systematic review from an A&E setting found only 23% of studies reported that teleconsultations were cost-effective [73].

Costs could be saved by improving access to specialist care in areas with limited local access to services. A systematic review of telepsychiatry concluded that cost savings would be made by doing just that, and they speculate that technology would become cheaper in the future [75]. A pilot study of teleconsultations in a rural dentistry setting found that up to £270 per patient appointment could be saved by the health service if it adopted teleconsultations to allow rural patients access specialist services [55]. In Scotland, a report on telehealth services found teleconsultations for a 10-week rehabilitation course could be delivered for 3% to 10% of the cost associated with an outreach model (where the therapist travels) or a centralized model (where the patient travels), with the savings primarily being delivered through reduced travel costs [91].

A service report from a well-established teleconsultation service in Airedale, which links an acute hospital with several care homes, reported setup and maintenance costs of £175,000 (US $223,938). However, factoring in costs from avoided A&E attendances and reduced nonelective admissions, the project is estimated to have saved £1,194,083 (US $1,529,939)—a saving of £6.82 (US $8.73) for £1 (US $1.28) invested [110].

In all, 2 RCTs, in different settings, comparing patient costs reported conflicting results. Jacklin et al [60], assessing teleconsultations in multiple outpatient specialties found those who took part in teleconsultations, saved an average of £19 (US $24.34) compared with face-to-face appointments. However, Noble et al [71], assessing its use in a minor injuries unit setting, found patient costs were nearly £15 (US $19) more.

#### Logistical and Operational Considerations

There were 16 studies that either assessed or commented on aspects relating to logistics or operational challenges. These included 3 RCTs [26,59,64], 1 case-control study [53], 2 qualitative studies [57,101], 2 mixed method studies of real-world teleconsultation services [102,105], 4 pilots [34,62,70,108], 1 cohort [66], 1 audit [95], 1 nonsystematic review [54], and 1 report [103]. There is considerable variation in the extent to which this is assessed and exactly what logistical element of interest was described.

Several studies make reference to consultation length, in which teleconsultations are as much as 4 times as long as their face-to-face equivalent [53,57,64]. However, several others found them to be shorter in length [34,102,105]. Williams et al [103] found no difference in consultation length but reports that by avoiding travel to peripheral clinics, clinicians were able to provide more emergency care with the time saved, thus maximizing their clinical efficiency. In older studies, there was often an intermediary, either a GP or another health care professional who would sit with the patient and establish the teleconsultation link [26,59,66]. The extent to which this disruption affects the service provision of the health care professional that is acting in this capacity is not described. In addition to normal duties, clinical staff have reported that they are often needed to *triage* those who might be suitable for teleconsultations [105]. Furthermore, clinicians may not have dedicated time to do teleconsultations, having to fit them around their normal outpatient schedules instead [59]. Benger et al [64] found that waiting times to access A&E advice were shorter for patients seen by teleconsultation than face-to-face consultation as they bypassed the normal admission processes—in essence skipping the queue.

Altering the way patients are seen can lead to improved operational efficiencies; Ditchburn et al [101] describe a service established to support patients undergoing peritoneal dialysis at home. By avoiding the need to travel to individual patient homes, staff reported that their time was used more efficiently as they were able to do other work on their computer while monitoring the patient via the video link. Nonattendance at hospital appointments is a source of lost revenue for health care providers and results in inefficient use of clinician time. By selectively choosing patient populations with high DNA rates, it is possible to achieve more operational efficiency. Morris et al [102], among a diabetic cohort, improved the DNA rate from 28% to 13%. Morey et al [108], among a prison population, describe a complete overhaul of a hepatitis C screening program pathway (along with other measures, teleconsultations were introduced), which led to a significant fall in DNA rates. These examples demonstrate clinical staff as drivers of change, but they can also be barriers to wider implementation [70].

Teleconsultations, as described by many of the studies included, can be seen as supporting a *hub and spoke* model of care, with district general hospital (DGH) *spokes* using teleconsultations to connect with more specialized *hub* hospitals. Agarwal et al [95] describe a telestroke *mesh* network of DGHs without a central *hub*, where out of hours stroke thrombolysis support was provided using telestroke rota shared across the region, thus reducing the frequency of a stroke physician’s *on-call* nights. Furthermore, such a model meant that thrombolysis care could continue without significant investment in staff and reorganization of thrombolysis care into a *hub and spoke* model.

The extent to which these services have been integrated into routine practice has been largely superficial. This means that small scale services were often provided with *ad hoc* support from IT departments rather than formal arrangements [101]. To provide a wider rollout of teleconsultations would require dedicated support from IT. Greenhalgh et al [105], through key stakeholder interviews, reported that NHS IT processes would require major changes to speed up the introduction of new technologies into practice.

In the NHS, hospital trusts are reimbursed through tariffs, often based on a *per-patient seen* basis, with different tariffs in place for face-to-face consultations and phone consultations. No such tariff existed for teleconsultations, which means that managers are often unwilling or unable to justify diverting the cost of such services from increasingly stretched clinical budgets [70,105].

## Discussion

This scoping review was aimed at assessing the extent of literature around UK-based teleconsultation patient interventions and the main outcome measures. The use of teleconsultations stretches back nearly 25 years, encompassing over 20 different specialties. Most of the specialties are represented in only a few articles and, though quality assessment of articles was not undertaken, pilots and case reports/series represent a significant proportion of that breadth. It is perhaps surprising to see that the decade between 1995 and 2005 accounts for two-thirds of the articles covered by this review, including all but 2 of the RCTs. The reasons behind this are not clear, but it may be a by-product of the challenging public finances since the 2008 economic recession. In that time, the NHS’s budget has faced a sustained period of constrained annual growth of 1.1% to 2.3%, compared with an average annual rise of 6% in the preceding years from 1996 to 2009 [111]. There was a narrowing of clinical focus during these years, perhaps to focus on where the need was most acute; the most enduring success of patient-facing teleconsultations in the United Kingdom is its use in acute stroke management, an intervention which was first reported in 2012 [92].

Nonetheless, the era in which most of these studies were carried out presents a number of problems for modern generalizability. Early studies used bespoke, expensive, complex, and cumbersome systems, which have now been largely superseded by the development of Web-based video calling technology such as Skype (Microsoft Corporation). This, coupled with the rise in smartphone use since 2007, means that videoconferencing technology is now in the pockets of millions of patients [112]. In a United Kingdom setting, however, relatively few studies have been done using this new technology.

### Technical Feasibility

The clarity of many older audiovisual connections was criticized—particularly by professionals [14,43], though patients were not universally satisfied that they could see and hear everything that was needed either [39]. The technology employed in these studies has now been superseded by Web-based platforms. Using these modern solutions does not prevent technical issues, and contingency plans need to be considered to overcome common problems, such as poor internet speed and lack of an audiovisual stream with Web-based solutions [105]. Although potentially prohibitive, these technical issues were usually rectifiable to allow continued operation of these services, but it does raise concerns that clinician time is being used inefficiently in such cases.

In total, 9% of the population (disproportionately older people) have never used the internet [113]. This, among other reasons outlined below, makes teleconsultation services unlikely to be accepted as a replacement to traditional care models and more likely that it should be offered as a choice.

### User Satisfaction and Experience

For the most part, patients seem to be satisfied with their experience of teleconsultations. Indeed, it seems that most teleconsultation interventions are aimed at improving aspects of the patient experience, such as convenience, rather than improving the experience of health care staff. They recognize its convenience and its utility when accessing specialist care in remote areas, or in time-sensitive matters. In some cases, patient satisfaction was higher than traditional clinic models, although as one author points out, this may be due to the increased accommodation provided to these patients for participation [31]. Avoidance of travel, although also convenient, may prove more pertinent; hospital-associated travel may cause stress in its own right, with 20% of older patients finding simply getting to and from appointments causes increased stress and anxiety [114].

It is interesting to note that among cancer patients, there was a preference for receiving *bad news* at home suggesting that even complex or challenging discussions may be had over teleconsultations. This is not a consistent finding internationally; patients in an acute medical setting had opposing views on receiving bad news over the video link. One patient in favor of such an approach stated, “If it was something earth-shattering, you could cry in your own bedroom and not have to worry, I mean driving from downtown and you’re upset or what-not....” But others were against this, explaining, “If the doctor were telling me I have a fatal disease or a disease that could be fatal, and I have to go into immediate serious care, probably better in-person” [115].

More is not always necessary or better as sometimes the telephone is sufficient. Therefore, it is important to explore if teleconsultations are needed to provide the intended benefit [61]. It should be noted that patients can be quite satisfied with their teleconsultation but still perceive face-to-face appointments to be the gold standard [107,109].

Among health care professionals, the view is more divided. Preconceptions about its utility from the perspective of the health care professional undoubtedly dictate the enthusiasm with which the service is promoted, in some cases, failing to even consider it as an option [105]. It is unlikely that a consensus will ever be so unanimous as to universally accept teleconsultations, and the wide variation of views on utility and acceptability means that they should only be offered as a choice and not a replacement to traditional models of care [76,107].

### Clinical Effectiveness

Teleconsultations appear to be a safe and effective way to assess and manage a variety of clinical situations. Clinical consensus, even within specialties, is not universal, however, and the types of consultations that are suitable are dependent on their complexity and physician comfort with the medium. Although physical examination is limited in teleconsultations, there are many examples in both inpatient and outpatient settings that demonstrate its utility. Neurological conditions and simple ophthalmological presentations such as strabismus could be safely diagnosed and managed.

The inability to perform some aspects of physical examination is likely, in some cases, to restrict its utility to more *routine* outpatient appointments. Among an inpatient, A&E, or acute stroke setting, the presence of a *proxy* examiner appears to be an effective way of overcoming this. Although proxy examiners (often GPs) were used in several outpatient-based studies, more recent outpatient studies assessed patients at home without a proxy present. The NHS is experiencing staff shortages, which are most acute in nursing (1 in 10 posts vacant) and general practice (1.6% decline in numbers) [116]. The use of proxy examiners is unlikely to be viable; therefore, the outcomes reported in such studies may not be replicable in today’s health service.

An interesting perspective on physical examination is that it has become a ritual, expected, and performed as tradition rather than clinical usefulness [117]. Novel technological solutions already allow certain physiological parameters—such as peak expiratory flow rate, heart rate/rhythm, and remote blood sugar levels—to be monitored remotely [118]. Digital stethoscopes can allow heart sounds to be transmitted via Bluetooth to a connected device [119], and smartphone ophthalmoscopes may be easier to master than direct ophthalmoscopy [120]. Wearable technology continues to develop, and solutions to other more nuanced aspects of physical examination may be developed in the future, however, for the time being, teleconsultations in outpatient settings are most likely to be confined to dialogue-based consultations where the need for rigorous physical examination is absent.

### Cost

The nature and method of assessment of cost were assessed in a heterogeneous way, which makes the comparison between studies speculative; however, it is clear that technology-associated costs have changed. Early studies used bespoke technological solutions with often prohibitive setup costs [26]. Technological advances mean that commercial teleconsultation services are fully scalable to the needs of the health care provider. Whereas many older studies almost universally found costs for the health care provider to be higher than the traditional model of care, more recent evidence from NHS Airedale’s experience shows that investment in a large-scale service can save significant costs by reducing unplanned admissions [96]. By reducing missed patient appointments, trusts can also make significant cost savings. Notwithstanding a few examples, real-world data on the financial implications of teleconsultations is lacking. Further in-depth case studies and service evaluations of established services are needed to accurately model the financial implications of teleconsultations.

For patients, the potential cost saving is more clear cut where travel and parking fees are only a part of the cost incurred. The true cost of patient time is likely to be much higher with one estimate putting the actual cost at £17.86 (US $22.89) per hour of travel, compared with just £1 (US $1.28) for a digital interaction [121].

### Logistical and Operational Considerations

Clinical trials have failed to replicate real-world operational challenges that such a service would create and is a distinct disadvantage to using a clinical trial methodology to assess the utility of digital technology in health care. The successful adoption of technology may be predicated on demonstrating safety and acceptability, but it will only survive in the real world if it can be integrated into existing health care pathways.

Several examples of real-world evaluations of working teleconsultation services have demonstrated that they can achieve meaningful reductions in DNA rates [102,108]. In one example, this required a complete overhaul of the existing clinical pathway, which was not fit for purpose [108]. Notwithstanding this example, a redesign of most clinical and administrative pathways would be a costly and an enormous logistical undertaking. In this, there is a disconnect between policies aimed at promoting more digital technology use and the real-world practicalities of establishing these services in busy and financially stretched hospital trusts. Embedding teleconsultations into routine clinical practice, in reality, has proven more complex than expected [105].

A key goal of the *Long-Term Plan* is to reduce the number of outpatient appointments, which have doubled to 120 million in the last decade. Through the use of technology, the NHS hopes to reduce this by a third over the next 5 years [4]. To achieve this, there is the promise of *central funding*, which trusts can access for technological improvements. It is not clear that this is ring-fenced in a way that will allow unencumbered access to service development funding. The expectation may be that these services will fund themselves through the anticipated £1 billion a year saving the outpatient reduction will achieve. As provided for in the National Tariff Program, trusts can opt to fund services on a block contract basis rather than a payment by results (PbR) basis. This would allow trusts more flexibility in how they engage with patients. A concern of this model is that unexpected service costs would not be reimbursed; however, PbR-based reimbursement has been criticized for the apparent incentivization of trusts to simply see more patients. NHS England has introduced a new digital tariff into the National Tariff payment system, which provides for reimbursement at 75% the rate of face-to-face consultations [122]. This should provide financial reassurance for trusts using a PbR model. Redesigning payment systems to be more flexible, perhaps to include elements of both, may be needed to overcome the diverse funding needs of the NHS [123].

### Where Do We Go From Here?

Well-funded, goal-orientated implementation of teleconsultations has been shown to be viable on a large scale in the NHS [96]. A number of important factors show that it is not known how replicable these results would be at a national level; NHS IT infrastructure is recognized to be stretched, and reluctance from health care professionals can stifle the growth of such innovations. Further investment is needed to address these issues [70,105].

Teleconsultations may not be suitable for every population. Therefore, teleconsultation services should be introduced gradually in a way that allows proper evaluation, with staff and patient feedback being used to fine-tune the pathway to suit local service needs and expectations. Routine clinical interactions are likely to represent the most pragmatic starting point for most services, but that is not to say that teleconsultations should be limited to such scenarios. To date, clinical interactions have been limited by the ability to perform examinations, but complex scenarios that involve a verbal exchange only, such as breaking bad news to patients with cancer, can be done effectively over a video link. Such interactions necessitate further investigation.

Although the *gold standard* of research methods, more RCTs are, arguably, not the correct way to find these answers. Finch et al [124], in an ethnographic study of telehealth integration into health care, found that participants felt that the RCT design conflicted with the dynamic nature of the health service environment. Participants saw greater value in pragmatic service evaluations that often produced results, which evaluators could use and—unconstrained by a rigorous trial protocol—they could adapt the service more readily to improve the project’s stability.

### Limitations

Scoping reviews are not intended to assess the quality of the literature included; therefore, the conclusions of this review are based on the existence of published research rather than the quality of it. Nevertheless, this scoping review provides a comprehensive, contemporary overview of the existing research on teleconsultations in a UK setting.

### Conclusions

Teleconsultations appear to be safe and effective in the right clinical situations. Where offered, it is likely that patients will be supportive of such measures, although they should only be offered as an option to support traditional care models rather than replace them outright. Health care staff should be encouraged and supported in using teleconsultations to diversify their practice. Health care organizations should consider developing digital technology strategy and implementation groups to assist health care staff in integrating technologically enabled care into routine practice. The introduction of new technologies should be assessed after a set period with service evaluations, including feedback from key stakeholders.

## Appendix

Multimedia Appendix 1 Summary of UK teleconsultation trials.

## Abbreviations

A&E: accident and emergency
DGH: district general hospital
DNA: did not attend
GP: general practitioner
IT: information technologies
NHS: National Health Service
PbR: payment by results
PRISMA: Preferred Reporting Items for Systematic Reviews and Meta-Analyses
RCT: randomized controlled trial
