# Supplementary material for: The Use of Patient-Facing Teleconsultations in the National Health Service: Scoping Review
Source: JMIR Med Inform. 2020 Mar 16;8(3):e15380. doi: 10.2196/15380 (PMC7105931; doi:10.2196/15380)
Supplement: Multimedia Appendix 1 [file medinform_v8i3e15380_app1.docx]

| Author | Year | Title | Specialty | Article type | No. of Participants | Outcome measure | Brief summary |
| --- | --- | --- | --- | --- | --- | --- | --- |

| Ball et al [9] | 1995 | A Comparison of Communication Modes in Adult Psychiatry | Psychiatry | Pilot | 12 | User acceptability and satisfaction | Feasibility study assessing the role of video consultations in acute psychiatry in 6 staff and 6 patients. Patients and staff found video consultations better than telephone consultations as both liked visual cues. |
| --- | --- | --- | --- | --- | --- | --- | --- |
| McLaren et al [10] | 1995 | An Evaluation of the Use of Interactive Television in an Acute Psychiatric Service | Psychiatry | Qualitative study | 105 | Clinical effectiveness | In all, 105 clinical interactions were studied. Videoconferencing was suitable for patient care and suggested telepsychiatry may become a major method of provision. |
| Siderfin et al [11] | 1995 | Telemedicine in the British Antarctic Survey Medical Unit | GP^a^ | Review | N/A^b^ | N/A | Reports the experience of the British Antarctic survey teleconsultation link and suggests similar benefits are achievable in Scotland. |
| Darkins et al [12] | 1996 | Point-to-Point Telemedicine Using the ISDN^c^ | Emergency | Pilot | 49 | Clinical effectiveness, cost | In all, 49 patients with minor injuries were managed successfully using teleconsultation. Cost of hardware prohibitive. |
| Harrison et al [13] | 1996 | The Future Role of Telemedicine at the Interface Between Primary and Secondary Care. | GP | Review | N/A | N/A | Focuses on the potential for the development of teleconsultation between primary care and secondary care. |

| Harrison et al [14] | 1996 | Can Telemedicine Be Used to Improve Communication Between Primary and Secondary Care? | Multiple | Pilot | 54 | User satisfaction, technical feasibility, and clinical effectiveness | Feasibility study assessing video consultations between primary care and secondary care. In all, 54 consultations. The main outcome measure was satisfaction with the consultation from both perspectives. The majority of staff patients were satisfied (>90% for both). However, 46% of consultants were critical of the audiovisual quality. Dermatologists especially felt it was not good enough to make an accurate diagnosis. |
| --- | --- | --- | --- | --- | --- | --- | --- |
| Jones et al [15] | 1996 | Teledermatology in the Highlands of Scotland | Dermatology | Pilot | 51 | Clinical effectiveness, user satisfaction | Pilot in 51 patients. Over half of the patients were able to be managed by video link alone. Patients were uncomfortable with it as a new consultation and would prefer it on follow-up. Concludes by suggesting a role for screening. |
| Kunkler et al [16] | 1998 | A Pilot Study of Teleoncology in Scotland | Oncology | Pilot | 8 | User satisfaction | Pilot set up to offer advice on nonclinic days, to complement the existing service. In all, 18 video conferences over 6 months with 8 patient-facing interactions. Patients and staff found it acceptable and were satisfied with the outcome (assessed by feedback questionnaire). |
| Curry et al [17] | 1998 | The Lancashire Telemedicine Ambulance | Emergency | Case report | 1 | Technical feasibility | The technical feasibility study demonstrated that a teleconsultation link is possible with mobile phone technology. |
| Loane et al [18] | 1998 | Patient Satisfaction With Real-Time Teledermatology in Northern Ireland | Dermatology | Cohort | 334 | User satisfaction | In all, 334 patients completeda 16-point questionnaire. High levels of user satisfaction and user comfort with the technology (85%). Time saver for patients. |
| Manchanda et al [19] | 1998 | Cognitive Behavior Therapy via Interactive Video | Psychiatry | Case report | 1 | Clinical effectiveness | Quasiqualitative case study exploring the feasibility of interactive video from cognitive behavioral therapy. Found no impediment to the clinical relationship. Unable to draw many other conclusions. |
| Freir et al [20] | 1999 | Telemedicine for Clinical Psychology in the Highlands of Scotland | Psychiatry | Review | N/A | N/A | Overview of the variety of applications being assessed in Scotland in relation to psychological assessment and therapy. |
| Harrison et al [21] | 1999 | Virtual Outreach: a Telemedicine Pilot Study Using a Cluster-Randomized Controlled Design | Multiple | RCT^d^ | 132 | Patient satisfaction | In all, 132 patients were randomized to the teleconsultation in their local health center or face-to-face clinic. User satisfaction higher in teleconsultation arm and significantly more time saved. |
| Patterson et al [22] | 1999 | Successful Management of Unexplained Coma by Telemedicine | Neurology | Case report | 1 | Clinical effectiveness | Successful management of a patient in a coma. The patient was diagnosed with status epilepticus via teleconsultation, which resulted in a treatment change and subsequent patient recovery. |
| Wan et al [23] | 1999 | Real-Time Remote Consultation in the Outpatient Clinic—Experience at a Teaching Hospital | Multiple | Pilot | 146 | User satisfaction | Describes a remote outpatient consultation service in 146 patients. Reports high levels of both patient (92% score >8/10 on Likert scale) and physician satisfaction (mean Likert scale score of 3.6/5). In all, 90% of patients would have another video appointment. |
| Wootton et al [24] | 1999 | Telemedicine and Isolated Communities: A UK^e^ Perspective | GP | Review | N/A | N/A | Concludes that teleconsultation evidence shows health services can deliver remote care, but most studies are international and not UK-based raising concerns about generalizability. |
| Loane et al [25] | 1999 | Patient Cost-Benefit Analysis of Teledermatology Measured in a Randomized Control Trial | Dermatology | RCT | 197 | Cost to patients | RCT from 2000. This looked at patient costs. Patients saved time and traveled less than those attending hospital. However, it was more time consuming for the GP and dermatologist. Concludes by saying real-time teledermatology is patient-friendly but not service-friendly. |
| Loane et al [26] | 2000 | A Comparison of Real‐Time and Store‐and‐Forward Teledermatology: A Cost-Benefit Study | Dermatology | RCT | 96 | Clinical effectiveness,  cost to health service, logistical and operational considerations | Patients were undergoing teledermatology via 2 methods (real-time and store-and-forward). In all, 55% versus 31% of patients were able to be discharged via real-time versus store-and-forward suggesting greater efficiency and diagnostic accuracy in real-time. However, real-time was more time consuming for GPs and dermatologists; therefore, the cost of real-time to the health care service was significantly higher £132.(US $169) vs £26.90 (US $34.50). It concludes by suggesting that store-and-forward is likely to be useful as a screening tool but that real-time may have a place in the right circumstances. |
| Craig et al [27] | 2000 | The Cost-Effectiveness of Teleneurology Consultations for Patients Admitted to Hospitals Without Neurologists on Site. 1: A Retrospective Comparison of the Case-Mix and Management in 2 Rural Hospitals | Neurology | Retrospective study | N/A | Cost | This study looks at a teleneurology service and compares the case-mix with another similar-sized hospital to assess if it is feasible to measure the effect of the teleconsultation service on care. No costs were evaluated. |
| Craig et al [28] | 2000 | Interactive Video Consultation is a Feasible Method for Neurological In-Patient Assessment | Neurology | Case-control | 25 | Clinical effectiveness | In all, 25 patients were assessed by video link and later seen face-to-face to validate the video assessments, acting as their own controls. High concordance of management between both. It concludes that it is practical to assess patients admitted with neurological symptoms to distant hospitals using teleconsultation, and this may result in more efficient use of in-patient resources. |
| Craig et al [29] | 2000 | A Pilot Study of Telemedicine for New Neurological Outpatient Referrals | Neurology | Case-control | 25 | Clinical effectiveness | Similar to the above article but in an outpatient setting. Concordance in 96% of cases in management between teleconsultations and face-to-face assessments. It suggests this may improve access to specialist care. |
| Graham et al [30] | 2000 | Could a Low-Cost Audiovisual Link Be Useful in Rheumatology? | Rheumatology | Pilot | 20 | Clinical effectiveness | It looks specifically at the diagnostic accuracy of both phone and video phone assessments. Accuracy was poor; 35% to 40%. Raises concerns about missed signs in 85% of cases and poor rapport over the video link. |
| Mair et al [31] | 2000 | Systematic Review of Studies of Patient Satisfaction With Telemedicine | Multiple | Systematic review | N/A | Patient satisfaction | An early review of patient satisfaction with teleconsultations. Included 7 UK trials; 2 in psychiatry, 3 in dermatology, 1 in oncology, and 1 multispecialty trial. Although generally well received by patients, the generalizability of the results was questioned due to the variety of unusual settings where teleconsultation was used (ie, prisons in the United States). It also criticized the special treatment participants received in minimizing inconvenience and usually being seen in person as well. Concludes by saying patient satisfaction could not be reliably confirmed on the available evidence. Furthermore, for the United Kingdom, 511/580 patients were in dermatology, with the remaining 69 spread across 4 pilots. |
| Haslam et al [32] | 2000 | Interactive Television for an Urban Adult Mental Health Service: The Guy's Psychiatric Intensive Care Unit Telepsychiatry Project | Psychiatry | Pilot | Data not available⸺ | Technical feasibility | Feasibility study in establishing a telepsychiatry program. |
| May et al [33] | 2000 | Telepsychiatry Evaluation in the North-West of England: Preliminary Results of a Qualitative Study | Psychiatry | Qualitative study | 35 | User satisfaction | In all, 22 patients and 13 doctors were interviewed after a video link consultation. Mixed views about the service. Patients saw the service as a means of obtaining additional expert advice. That is, improving access to specialist care. Staff worried the doctor-patient relationship might be affected. Recognition that the video link modified normal interaction. |
| Salmon et al [34] | 2000 | Telemedicine Use in 2 Nurse-Led Minor Injuries Units | Emergency | Pilot | 624 | Clinical effectiveness, logistical and operational considerations | Reports experience from the first year of 2 teleconsultation links in minor injury units, a total of 624 uses. The use of teleconsultations was 2.3% in one and 5.2% in another, no clear reason found for the higher usage. Reports consultation time of 3.5 min, which seems acceptably short. |
| Tachakra et al [35] | 2000 | How Do Teleconsultations for Remote Trauma Management Change Over a Period of Time? | Emergency | Retrospective study | 300 | Clinical effectiveness | A 4-year follow up of a minor injury unit service. Looked at the proportion of cases being seen by teleconsultation in 1999 versus 1996. As a proportion, teleconsultations fell from 5.6% to 3.8% of attendance. Reasons given were radiograph interpretation training meant fewer teleconsultations were required by nurses who were able to work more independently. |
| Tachakra et al [36] | 2000 | Supervising Trauma Life Support by Telemedicine | Emergency | Case series | 15 | Technical feasibility,  clinical effectiveness | Feasibility study assessing if a video link could be used to assess a major trauma case. In all, 15 simulated casualties replicated the ATLS^f^ scenarios for trauma training. Although difficulties were reported with camera view and having to use a proxy examiner, the paper concludes that physicians were confident in most aspects of care and that major trauma supervision by video link is feasible. |
| Wootton et al [37] | 2000 | Transfer of Telemedical Support to Cornwall From a National Telemedicine Network During a Solar Eclipse | Emergency | Pilot | 91 | Technical feasibility | A popup teleconsultation support service to assist with an expected peak in attendance during an eclipse in Cornwall. Minor injury units in Cornwall were linked to other A&E^g^ departments in the United Kingdom. In total, 2045 patients were seen, with 93 teleconsultation calls for 91 patients (4.6% of total). The authors conclude that teleconsultation is feasible on a national scale. |
| Beach et al [38] | 2001 | Evaluating Telemedicine in an Accident and Emergency Setting | Emergency | Protocol | 152 | User satisfaction, technical feasibility, cost | Protocol for a hub and spoke model of care with a larger A&E department providing specialist care via video link will aim to assess satisfaction, technical feasibility, and cost. |

| Bose et al [39] | 2001 | The Use of Telepsychiatry in the Brief Counselling of Nonpsychotic Patients from an Inner-London General Practice | Psychiatry | Pilot | 13 | Technical feasibility, patient satisfaction | Feasibility study assessing if counseling can be carried out via videoconferencing sessions. In total, 13 patients took part, and 93% reported that they would use it again if offered, and most (>75%) could see and hear what they needed. |
| --- | --- | --- | --- | --- | --- | --- | --- |
| Chua et al [40] | 2001 | Randomized Controlled Trial of Telemedicine for New Neurological Outpatient Referrals | Neurology | RCT | 168 | Clinical effectiveness, patient satisfaction | Aimed to test the hypothesis that teleconsultation for new patient referrals to neurological outpatients is as efficient and acceptable as conventional face-to-face consultation. Although diagnostic categories were similar, patients in the teleconsultation group had significantly more investigations. There was no difference in the number of drugs prescribed, and patient satisfaction was the same. Concerns about confidentiality and embarrassment in the teleconsultation arm were reported. Concludes that although it is feasible, teleconsultation generates more investigations and is less acceptable than face-to-face examination. |
| Chua et al [41] | 2001 | Cost Implications of Outpatient Teleneurology | Neurology | RCT | 141 | Cost | Cost analysis of theRCTby Chua et al, teleconsultations were more expensive than conventional appointments;£72 (US $92.26) vs £49 (US $62.79. |
| Leggett et al [42] | 2001 | Telerheumatology⸺Diagnostic Accuracy and Acceptability to Patient, Specialist, and General Practitioner | Rheumatology | Case-Control | 100 | Clinical effectiveness, user satisfaction | Looked at the diagnostic accuracy and acceptability of telephone and video consultations in rheumatology OPD^h^. Each case acted as its own control. Video consultations were accurate in 97% of cases and were acceptable to all parties involved. |
| McCullough et al [43] | 2001 | Viability and Effectiveness of Teletherapy for Preschool Children With Special Needs | Pediatrics | Pilot | 4 | Clinical effectiveness, technical feasibility, satisfaction, cost | Assessed if carers could be educated in developing their child’s communication skills in a special needs population. Parents and therapists reported improved communication skills in children. User satisfaction was high. Audio quality and sound lag were raised as concerns. Expensive to set up (£1795 per home). |
| Rayner et al [44] | 2001 | Subspecialty Adnexal Ophthalmological Examination Using Telemedicine | Ophthalmology | Case-Control | 17 | Clinical effectiveness | A case-control study with patients acting as their own control. Assessed the diagnostic accuracy of eyelid and orbit conditions via video link. The Investigators report that uncomplicated ptosis was accurately assessed but more complex eye conditions were not, raising concerns about its usefulness. |
| Simpson et al [45] | 2001 | The Provision of a Telepsychology Service to Shetland: Client and Therapist Satisfaction and the Ability to Develop a Therapeutic Alliance | Psychiatry | Pilot with a qualitative component | 10 | User Satisfaction | This pilot assessed the feasibility and acceptability of a remote clinical psychology service. Patients were interviewed to assess satisfaction and acceptability. Most users were highly satisfied and felt able to develop a positive therapeutic relationship. |
| Tachakra et al [46] | 2001 | Depth Perception in Telemedical Consultations | Emergency | Descriptive study | 235 | Technical feasibility | Reports coping mechanisms for dealing with problems with depth perception over an audiovisual link. Depth perception judged to be <90% in all cases but improved >90% with coping strategies in 42% of cases. Coping mechanisms can improve depth perception problems but not eliminate them. |
| Tachakra et al [47] | 2001 | Avoiding Artificiality in Teleconsultations | Emergency | Descriptive study | 33 | User experience | Reports methods of reducing the feeling of artificiality that teleconsultation interactions can create. Done in an artificial simulated clinical scenario rather than real life, that is, artificial by design. |
| Brebner et al [48] | 2002 | Evaluation of a Pilot Telemedicine Network for Accident and Emergency Work | Emergency | Pilot | 402 | Clinical effectiveness | Descriptive 15-month pilot between *hub and spoke* A&E units in Scotland. In all, 1998 videoconferencing calls were made, with 402 for clinical consultations. In total, 89% of patients were managed without the need for transfer aided by the use of a teleradiology link. Formed the basis of a government grant to expand the service. |
| Dawson et al [49] | 2002 | The Role of Telemedicine in The Assessment of Strabismus | Ophthalmology | Case-control | 30 | Clinical effectiveness | In total, 80% of strabismus cases were accurately diagnosed via a teleconsultation link, with a further 17% having some concordance. Concludes that strabismus can be accurately assessed by a teleconsultation link. |
| Harley et al [50] | 2002 | The Use of Videoconferencing to Enhance Tertiary Mental Health Service Provision to the Island of Jersey | Psychiatry | Pilot | 5 | User satisfaction | A 6-month pilot of telepsychiatry service in an island setting. In all, 5 teleconsultations done. Universal satisfaction with the concept and implementation. |
| MacFarlane et al [51] | 2002 | The Benefits of a Qualitative Approach to Telemedicine Research | Multiple | Qualitative study | 69 | User satisfaction/experience | A multifaceted qualitative study using semistructured interviews with 30 patients, 24 GPs, and 15 specialists. Multiple focus groups and analysis of consultation recordings. Concludes that staff and patients have different perceptions of how video consultations went. |
| Martin et al [52] | 2002 | Using Commercially Available Technology to Assist in the Delivery of Person-Centered Health and Social Care | Multiple | Pilot | ⸺ | User satisfaction/experience | The first study to look at videoconferencing with patients at home. Clients report feeling less isolated at home and more secure. Used as a basis for establishing a telecare service, though not necessarily using videoconferencing. |
| McLaren et al [53] | 2002 | The North Lewisham Telepsychiatry Project: Beyond the Pilot Phase | Psychiatry | Case-control | 31 | Clinical effectiveness, logistical and operational considerations | Treatments took longer to complete via teleconsultation, with nearly half of patients still on treatment at completion versus 25% of face-to-face consultations. |
| Patterson et al [54] | 2002 | Teleneurology in Northern Ireland: A success | Neurology | Review | N/A | Technical feasibility, cost, user satisfaction, clinical effectiveness, logistical and operational considerations | Describes the successful implementation of a remote teleconsultation service in Northern Ireland based on earlier discussed studies. Comments on the struggles to get traditional research bodies to fund projects and the need to modify individual consultation techniques for teleconsultations. |
| Scuffham et al [55] | 2002 | An Economic Evaluation of the Highlands and Islands Teledentistry Project | Dentistry | Pilot | 25 | Cost | Set in a rural island location. Cost minimization was assessed when patients were assessed by teleconsultation. Between £154 and £270 could be saved on travel costs by teleconsultation uptake. |
| Simpson et al [56] | 2002 | Video-Hypnosis—The Provision of Specialized Therapy via Videoconferencing | **P**sychiatry | Pilot with qualitative feedback | 11 | User satisfaction, technical feasibility | Assessment of if hypnosis was feasible via teleconsultation. All participants were satisfied with telehypnosis and indicated that they would like further sessions. The audiovisual link was adequate. |
| Tachakra et al [57] | 2002 | Social Presence in Telemedicine | Emergency | Qualitative Study | 60 | User experience/satisfaction, logistical and operational considerations | In all, 30 patients each took part in a video or face-to-face clinic appointment, and interactions were observed. Teleconsultations were nearly 4 times longer than face-to-face consultations. Patients seemed empowered to ask more questions via teleconsultations, and turn-taking was more common too. Doctors seemed to take greater care in a teleconsultation to achieve coordination of beliefs with the patient than in a face-to-face consultation. |
| Tachakra et al [58] | 2002 | Four Years' Experience of Telemedicine Support of a Minor Accident and Treatment Service | Emergency | Retrospective study | 2032 | Clinical effectiveness | Reports 4 years’ experience of a teleconsultation service. In total, 3.6% of all cases seen in that time were via teleconsultation, 46% of these were to review x-rays, and 15% concerned simple fracture management, and >90% of cases were managed without transfer suggesting care was managed safely locally. With time, the referral rate to GP/main hospital had halved suggesting time improved confidence in the local service. |
| Wallace et al [59] | 2002 | Joint Teleconsultations (Virtual Outreach) Versus Standard Outpatient Appointments for Patients Referred by Their General Practitioner for a Specialist Opinion: A Randomized Trial | Multiple | RCT | 2094 | Clinical effectiveness, user satisfaction, logistical and operational considerations | Comparison of virtual outreach to conventional outpatients. 1:1 randomization. More patients in the virtual outreach group than the standard group were offered a follow-up appointment (502, 52%, vs 400, 41%), Fewer tests and investigations were ordered in the virtual outreach group by an average of 0.79 per patient Patients’ satisfaction (analyzed per protocol) was greater after a virtual outreach consultation than after a standard outpatient consultation. There was increased follow-up for the virtual outreach group mainly based on location (Shrewsbury) and specialty (orthopedics and ENT^i^ surgery). Concludes that teleconsultation can improve efficiency and reduce investigations, but specialties will vary based on need. |
| Jacklin et al [60] | 2003 | Virtual Outreach: Economic Evaluation of Joint Teleconsultations for Patients Referred by Their General Practitioner for a Specialist Opinion | Multiple | RCT | 2094 | Cost | Cost benefit analysis of the RCT by Wallace et al. Teleconsultations were not cost-neutral to the NHS^j^. Mean difference of £99 to £108 (about 15%) higher in the virtual outreach group. Patients saved time and money, an average of £19 (including loss of productive time). |
| Cargill et al [61] | 2003 | Telecare Support for Patients Undergoing Chronic Peritoneal Dialysis | Nephrology | RCT | 6 | Technical feasibility, user satisfaction | Small RCT looking at benefits of telecare in peritoneal dialysis. Included the use of a video phone, which was received with mixed results. Families saw the benefit of being seen at home, but issues arose with lag and image quality. The telephone was seen as more important than videophone. |
| Ferguson et al [62] | 2003 | Minor Injuries Telemedicine | Emergency | Pilot | 407 | Clinical effectiveness, logistical and operational considerations | Looks at transfer rates from a *spoke* to a *hub*. Concludes that transfer rates go down as familiarity with technology increases suggesting benefits may be greater beyond the pilot phase. |
| Palombo et al [63] | 2003 | An Evaluation of a Telemedicine Fracture Review Clinic | Orthopedics | Pilot | 50 | Clinical effectiveness | Assessed safety of management and concluded that patients could be safely managed by teleconsultation. |
| Benger et al [64] | 2004 | The Safety and Effectiveness of Minor Injuries Telemedicine | Emergency | RCT | 600 | Clinical effectiveness, logistical and operational considerations | Interesting trial. Patients were seen in the A&E department by 3 different methods, each blinded to the others’ assessment. Treatment plans were written independently by each physician (teleconsultation consultant, onsite specialist, and GP). Patients were then randomized to receive one of these treatment plans on 1:1:1 basis. This allowed direct assessment of safety and discrepancies. The gold standard was review a week later with radiologist reports of radiographs and patients seen face-to-face by the specialist. All patients had this follow-up against which their original treatment plan was compared. In total, 73 discrepancies identified with 12 important overtreatments and 11 important undertreatments, but these were evenly spread across the 3 assessment arms. Concludes teleconsultation is not inferior to other methods of assessment and is safe. |
| Brebner et al [65] | 2004 | Evaluation of an Accident and Emergency Teleconsultation Service for North-East Scotland | Emergency | Pilot | 1392 | Clinical Effectiveness | Reports a 1-year experience from a teleconsultation model in Scotland. In total, 77% of patients were able to be managed locally without transfer. |
| Craig et al [66] | 2004 | A Cohort Study of Early Neurological Consultation by Telemedicine on The Care of Neurological Inpatients | Neurology | Cohort | 111 | Clinical effectiveness, logistical and operational considerations | Length of stay was dependent only on age. However, there was a small nonsignificant reduction in bed stay length, which the authors conclude is important and probably reflects the more timely intervention of specialist opinions. There was no difference in the secondary outcomes compared with the unstudied population. |
| Grant et al [67] | 2004 | Telemedicine in the British Antarctic Survey | Emergency | Review | N/A | Technical feasibility | Describes the evolution of teleconsultation over 50 years in the Antarctic and how it has evolved from telephone, fax, and email to now include real-time teleconsultations. |
| Leonard et al [68] | 2004 | The Development and Evaluation of a Telepsychiatry Service for Prisoners | Psychiatry | Protocol | N/A | Cost, user satisfaction | Describes the establishment of a telepsychiatry service and a study protocol arising from it, which would involve a mixed method analysis of cost and satisfaction. |
| Coelho et al [69] | 2005 | An Assessment of the Efficacy of Cancer Genetic Counseling Using Real-Time Videoconferencing Technology (Telemedicine) Compared with Face-to-Face Consultations | Genetics | Case-control | 37 | Clinical effectiveness, user satisfaction | Patients were assigned an arm depending on geographical location (not random). Aimed to evaluate counseling over video conference link. A significant reduction in cancer-related anxiety levels and high satisfaction levels were reported in both groups. |
| Grealish et al [70] | 2005 | Telemedicine in a Child and Adolescent Mental Health Service: Participants’ Acceptance and Utilization | Psychiatry | Pilot | 5 | User satisfaction, Logistical & operational considerations | Over 2 years, 5 patients took part in 20 teleconsultations. User satisfaction was high; however, there was reluctance and resistance from staff who were not keen to use it, which suggests child and adolescent mental health is not suitable for teleconsultations. |
| Noble et al [71] | 2005 | A Cost-Consequences Analysis of Minor Injuries Telemedicine | Emergency | RCT | 253 | Cost | Associated with Benger et al’s RCT on minor injury unit teleconsultation assessment. Costs were higher in the teleconsultation arm to both the NHS (£78.61 vs £39.15) and patients (£58.24 vs £43.95) in a GP-supported minor injuries unit setting. |
| Soopramanien et al [72] | 2005 | Using Telemedicine to Provide Postdischarge Support for Patients With Spinal Cord Injuries | Rehabilitation | Case report | 1 | Clinical effectiveness | Case of potential benefit in a patient with a spine injury. Better access to specialist care. |
| Brebner et al [73] | 2006 | Accident and Emergency Teleconsultation for Primary Care⸺A Systematic Review of Technical Feasibility, Clinical Effectiveness, Cost-Effectiveness, and Level of Local Management | Multiple | Systematic review | N/A | Technical feasibility, clinical effectiveness, cost | In total, 2 RCTs available. Most were pilots. All studies reported to be technically feasible. In total, 97% were reported as clinically effective, 48% gave figures on local management, which averaged 76% across the studies. Only 23% showed evidence of cost-effectiveness. |
| Chambers et al [74] | 2006 | Longitudinal Trends in Quality of Life After Starting Home Parenteral Nutrition: A Randomized Controlled Study of Telemedicine | Nutrition | RCT | 30 | Clinical effectiveness | Telephone versus teleconsultation follow-up of home parenteral nutrition support. Teleconsultation had absolutely no bearing on the outcome measure. |
| Norman et al [75] | 2006 | The Use of Telemedicine in Psychiatry | Psychiatry | Systematic review | N/A⸺ | Clinical effectiveness, technical feasibility, cost | Review of telepsychiatry utility for a UK setting. Although it aims to assess telepsychiatry in the United Kingdom, most studies included are based outside the United Kingdom. It concludes that telepsychiatry can improve access to mental health services, especially in rural areas, and that technology advances are making it more cost-effective and reliable. |
| Harrison et al [76] | 2006 | Patients’ Perceptions of Joint Teleconsultations: A Qualitative Evaluation | Multiple | Qualitative study | 28 | User satisfaction/experience | Qualitative interview study linked to the virtual outreach RCT from 2000. There was a wide variety of views from patients as to the utility and acceptability of teleconsultation. Most saw it as convenient and time-saving but felt where examination may be necessary, then face-to-face consultation would be better. Opinions were also that virtual appointments should be a choice and not the only option for those who wish to be seen face-to-face. |
| Weatherburn et al [77] | 2006 | An Assessment of Parental Satisfaction With Mode of Delivery of Specialist Advice for Pediatric Cardiology: Face-To-Face Versus Video Conference | Pediatrics | Pilot | 100 | User satisfaction | In total, 20 patients had teleconsultations, 56 had outreach clinics, and 24 had a specialist center follow-up. Satisfaction high across all modes. Those who had teleconsultations felt it would save money but were less enthusiastic about replacing face-to-face appointments with them. |
| Dowie et al [78] | 2007 | Telemedicine in Pediatric and Perinatal Cardiology: Economic Evaluation of a Service in English Hospitals | Pediatrics | Case-control | 504 | Clinical effectiveness, cost | In total, 117 patients had teleconsultation appointments versus 387 conventional appointments. Clinical outcomes are difficult to compare here, as teleconsultation patients were generally healthier. Teleconsultations were more expensive (£411 vs £277). |
| Singh et al [79] | 2007 | Accuracy of Telepsychiatric Assessment of New Routine Outpatient Referrals | Psychiatry | Single-cluster, balanced crossover, blind study | 37 | Clinical effectiveness | There were substantial intermethod agreements on all the major components of evaluation except on the risk assessment scale where there was only weak agreement. Telepsychiatric assessment is a dependable method of assessment with a high degree of accuracy. |
| Mair et al [80] | 2008 | More Patients With Minor Injuries Could Be Seen by Telemedicine | Emergency | Retrospective study | 112 | Clinical effectiveness | Looks at a 6-month retrospective period. Reviewed 112 cases that were referred to the main unit. Suggests greater teleconsultation experience results in fewer referrals. |
| Mair et al [81] | 2008 | Telemedicine via Satellite to Support Offshore Oil Platforms | GP | Pilot | N/A | User satisfaction | In total, a 9-month pilot of a teleconsultation with an offshore oil rig. Three physicians were satisfied with each clinical encounter. |
| Mair et al [82] | 2008 | Feasibility of a Telemedicine *Booth* for Supporting Remote Care in Scotland | GP | Pilot | 238 | Technical Feasibility | Assessment of a *telebooth* for teleconsultations. Participants felt it would allow better access to specialist advice. |
| Miller et al [83] | 2008 | The Delivery of a Minor Injuries Telemedicine Service by Emergency Nurse Practitioners | Emergency | Audit | 788 | Clinical effectiveness | A case review showed that overall, nurse practitioners were considered capable of treating 470 of 788 new presentations (60%). If children under 14 years of age and shoulder injuries were excluded, this figure rose to 84%. |
| Morgan et al [84] | 2008 | Home Videoconferencing for Patients With Severe Congenital Heart Disease Following Discharge | Pediatrics | RCT | 27 | Clinical effectiveness | Randomized to telephone or video conference follow-up in a Congenital Heart Diseasepediatric group. Satisfaction was higher in the video conference group and was found to be considered more beneficial. The video group was found to have a statistically significant benefit in reducing anxiety levels over the phone group. |
| Styles et al [85] | 2008 | Service Users' Acceptability of Videoconferencing as a Form of Service Delivery | Speech and language | Pilot | 12 | User satisfaction | In total, 88% were satisfied with the video conference assessment session and a 95% satisfaction with review video conference sessions. Clients were the most positive whereas the speech and language therapists were the most critical. |
| Webster et al [86] | 2008 | Provision of Telehealth to the Scottish Police College | Multiple | Pilot | 192 | Technical feasibility, clinical effectiveness | Feasibility study in a Scottish police college. Only half of the patients were seen by teleconsultation due to technical issues. In all, 76% of those seen were managed without an onward referral. Concluded that greater reliability of the technology would have made it feasible for the police college. |
| Wilkinson et al [87] | 2008 | A Feasibility Study of Home Telemedicine for Patients With Cystic Fibrosis Awaiting Transplantation | Respiratory | RCT | 11 | Clinical effectiveness | Set in a terminally ill cystic fibrosis population. In total, 11 completed the baseline assessment, and 7 completed the study protocol. No difference between groups’ anxiety levels or other outcomes. Likely affected by small numbers. The consultation group did value the service, however. |
| Howell et al [88] | 2009 | Delivering the Lee Silverman Voice Treatment (LSVT) by Web Camera: A Feasibility Study | Speech and language | Pilot | 3 | Clinical Effectiveness | Assessment of the feasibility of delivering a specialized speech therapy over a video link. Successful trial in 3 patients. Improves access to specialist therapy. |
| Pappas et al [89] | 2009 | The Opening Phase of Telemedicine Consultations: An Analysis of Interaction | Multiple | Qualitative study | 10 | User experience | Analysis of 10 consultations via video link and uses conversation analysis to explore the differences between it and standard face-to-face consultations. The Investigators reportthat teleconsultation change the doctor-patient dynamic. Suggests that staff training may be important to correct this. |
| Harvey et al [90] | 2010 | Eleven Years of Experience With Low-Bandwidth Telemedicine in a Nurse-Led Rural Clinic in Scotland | GP | Descriptive study | 646 | Clinical effectiveness | In total, 11-year experience of teleconsultation in a rural GP practice. Teleconsultations were successfully used for a variety of health matters including mental health and physical health. Suggests organizational decisions will determine viability. |
| Hill et al [91] | 2010 | Telerehabilitation in Scotland: Current Initiatives and Recommendations for Future Development | Rehabilitation | Review | N/A | Cost | A wider review of teleconsultation services in Scotland. Reports on the use of teleconsultations in conjunction with other telecare methods in rehabilitation. Suggests that telehealth could save significant costs over a 10- week rehab course⸺£246 versus £7728 (outreach) versus £2297 (centralized). |
| Chowdhury et al [92] | 2012 | Telemedicine Versus Face-to-Face Evaluation in the Delivery of Thrombolysis for Acute Ischemic Stroke: A Single Centre Experience | Stroke | Retrospective study | 45 | Clinical effectiveness | Reports no difference in outcome measures in patients managed by a teleconsultation link versus face-to-face consultation and suggests this is a safe and feasible method for out-of-hours thrombolysis care. |
| Gibson et al [93] | 2013 | Introducing Telemedicine in Acute Stroke: Patients' and Carers’ perspectives | Stroke | Qualitative | 24 | User satisfaction/experience | Describes the development of a standardized teleconsultation toolkit for acute stroke with qualitative feedback from patients and carers. Findings included: Positive opinions of the value and importance of teleconsultation; willingness to be involved in the teleconsultation, confidence in the skills of bedside staff, and decision-support providers. |
| Hill et al [94] | 2013 | The first 100 Thrombolysis Cases in a Novel Scottish Mesh Telestroke System | Stroke | Audit | 100 | Clinical effectiveness | Novel mesh telestroke service in Scotland linking 3 units to provide cross-cover. Before establishment, many patients waited 70 min to transfer to a tertiary center. The new service had comparable outcome measures as national averages and compared well with other new stroke services. |
| Agarwal et al [95] | 2014 | Thrombolysis Delivery by a Regional Telestroke Network⸺Experience From the United Kingdom National Health Service | **Stroke** | Audit | 142 | Clinical effectiveness, logistical and operational considerations | First years’ experience of a telestroke network in the East of England. Described as a *horizontal* model rather than a *hub and spoke* model in 7 equally sized DGHs^k^. This model reports teleconsultation use of over 50%⸺one of the highest rates in Europe and comparable outcomes to national averages suggesting a feasible model of care for similar geographic locations. |
| Hex et al [96] | 2015 | Telemedicine in Care Homes in Airedale, Wharfedale, and Craven | Care of the elderly | Service Report | N/A | Clinical effectiveness, cost | Report from an established teleconsultation network in the United Kingdom, which provides remote care to regional care homes. Care homes with teleconsultation showed a greater reduction in costs and admissions over nonteleconsultation care homes. Costs were reduced by 39% compared with 30% in nonteleconsultation care homes suggesting a potential for greater cost savings in a wider role out of care. |
| Murphie et al [97] | 2015 | Telemedicine Sleep Clinic Review⸺ Patient Acceptability Survey | Respiratory | Pilot | 60 | User satisfaction | Patients reported satisfaction with teleconsultations for sleep medicine reviews. Also reported a reduction in miles traveled and reduced carbon footprint. |
| Gibson et al [98] | 2016 | *It Was Like He was in the Room With Us*: Patients' and Carers’ Perspectives of Telemedicine in Acute Stroke | Stroke | Qualitative study | 29 | User Satisfaction/Experience | Assessed patient and carer perceptions of telestroke in both a live setting and mock setting. Generally well received. Patients see the value in teleconsultation when face-to-face consultation is not possible. It is a compromise that may be improved by good staff training and communication. |
| Ranta et al [99] | 2016 | International Telestroke: The First Five Cases | Stroke | Case series | 5 | Clinical effectiveness | Report of a case series of international telestroke between Scotland and New Zealand. This case series suggests that avoiding the challenges of nighttime coverage can be achieved by using international time zones as an advantage. |
| Bagot et al [100] | 2017 | Integrating Acute Stroke Telemedicine Consultations Into Specialists' Usual Practice: A Qualitative Analysis Comparing the Experience of Australia and the United Kingdom | Stroke | Qualitative study | 11 | User experience/satisfaction | A study looking at the variation of experiences between 2 countries’ telestroke networks (United Kingdom and Australia). Although similarities were found between interviewees in describing the value and concerns of telestroke, clinically Physicians differed on suggestions for improvements⸺the United Kingdom focused on governance and policy, consistent with the risk- adverse NHS and the Australians focused on work practice changes. |
| Ditchburn et al [101] | 2017 | Renal Telemedicine Through Video-As-A-Service Delivered to Patients on Home Dialysis: A Qualitative Study on the Renal Care Team Members’ Experience | Nephrology | Qualitative study | 10 | User satisfaction/experience, logistical and operational considerations | Describes the experience of a teleconsultation service to support home hemodialysis and uses qualitative methods with staff to gain their perspectives on the service. Staff felt teleconsultation provided a number of benefits such as improved efficiency, saves travel time, and improved job satisfaction. |
| Morris et al [102] | 2017 | Webcam Consultations for Diabetes: Findings From Four Years of Experience in Newham | Endocrinology | Mixed method | 480 | Clinical effectiveness, user satisfaction/experience, logistical and operational considerations | Over 4 years, 480 video appointments were held. DNA^l^ rate was 13% (vs 28% for the main clinic). Hemoglobin A_1c_ improvement was seen in video users but not statistically significant. Consultation time was found to be significantly shorter (9 min vs 25 min). Patients found it convenient and thought it delivered good care. Staff felt it was not appropriate for all consultations. Patients also reported feeling awkward and uncomfortable at times via video link. Results suggest efficiency saving can be seen in both cost and time, but further studies on perceptions of the staff were needed. |
| Williams et al [103] | 2017 | The Use of Telemedicine to Enhance Secondary Care: Some Lessons From the Front Line | Multiple | Service Report | N/A | Logistical and operational considerations | Report on the establishment of a multispecialty teleconsultation service in Wales. It has not yet been assessed, but plans are at hand to do so. Describes some barriers and tips to set up a service⸺Use patient satisfaction questionnaires, consider only using it with follow-up patients. Consider if an examination is important or not. |
| Freed et al [104] | 2018 | Telemedicine: Is it Really Worth it? A Perspective From Evidence and Experience | Multiple | Summary of systematic review | N/A | Cost | The authors aim to answer *Is an investment in teleconsultation worth it?* and *How do I make a teleconsultation implementation work?* with this review. Concludes that RCT data are confusing but that it is worth investing in telemedical initiatives. |
| Greenhalgh et al [105] | 2018 | Real-World Implementation of Video Outpatient Consultations at Macro, Meso, and Micro Levels: Mixed Method Study | Multiple | Mixed method | 107 | Technical feasibility, user satisfaction/experience, clinical effectiveness, logistical and operational considerations | Complex, multilevel mixed method study aiming to define good practice and inform the implementation of video consultations in outpatients. Set in 2 specialties (diabetes and cancer surgery). Video consultations appeared safe but not universally popular. Video consultations work better when the doctor-patient relationship was already established. Establishing video outpatient services in a busy NHS acute hospital setting was complex and time-consuming. Uptake in clinics was small (2%-22%, highest in cancer surgery) Consultations were being undertaken remotely. In the remainder, clinicians chose not to participate, or video consultations were considered impractical, technically unachievable, or clinically inadvisable. Technical challenges were typically minor but potentially prohibitive. Patients in the cancer surgery group felt they would prefer to receive bad news at home rather than face a long journey home after. |
| O’Connell et al [106] | 2018 | Video Clinics Versus Standard Face-To-Face Appointments for Liver Transplant Patients in Routine Hospital Outpatient Care: Study Protocol for A Pragmatic Randomized Evaluation of myVideoClinic | Transplant | Protocol | 180 (planned) | User satisfaction, cost | Study protocol for a parallel 2-arm statistician blinded randomized evaluation of video clinics in a liver transplant setting. Aims to assess multiple aspects of video clinic use with questionnaire-based feedback for satisfaction and quality of life. Economic assessment based on a cost consequence analysis for the NHS and patients. Qualitative feedback to be gathered from 14 patients/carers and 14 staff. |
| Haig-Ferguson et al [107] | 2018 | *It's Not One Size Fits All*: the Use of Videoconferencing for Delivering Therapy in a Specialist Pediatric Chronic Fatigue Service | Pediatrics | Qualitative | 27 | User experiences/satisfaction | In total, 12 patients from a pediatric setting took part in cognitive behavior therapy by teleconsultations, 6 parents took part in interviews, and 9 staff took part in a focus group. Majors themes: 1. Challenges/concerns: Privacy and personal connection concerned participants. 2. Benefits: Convenient, accessible, and the screen can help people to *open up* 3. Treatment: face-to-face consultation considered superior by participants, and teleconsultations should support rather than replace face-to-face care. Concludes that teleconsultations should be offered as an option within a package of care rather than a replacement. |
| Morey et al [108] | 2018 | Increased Diagnosis and Treatment of Hepatitis C in Prison by Universal Offer of Testing and Use of Telemedicine | Hepatology (Prison inmates) | Pilot | 80 | User satisfaction, clinical effectiveness, logistical and operational considerations | Teleconsultations were part of a wider project to increase the detection and treatment of blood-borne viruses in prison. In total, 80 inmates took part in follow-up teleconsultations. Satisfaction rates were high among inmates, and known clinical outcomes in participants were better than controls. Concludes that universal blood-borne virus testing in inmates with teleconsultations can improve testing, diagnosis, and treatment of the hepatitis C virus. |
| Gilbert et al [109] | 2018 | What is the Acceptability of Real-Time 1:1 Videoconferencing Between Clinicians and Patients for a Follow-Up Consultation for Multidirectional Shoulder Instability? | Orthopedics | Qualitative | 21 | User experience/Satisfaction | Explores the acceptability of teleconsultations in a follow-up setting. In total, 13 patients (7 who chose teleconsultations) and 8 staff were interviewed. Not universally acceptable but beneficial to avoid the pain associated with traveling (related to shoulder injury). Face-to-face still viewed by many as the gold standard as physical examination felt important by some staff. |

^a^GP: general practitioner.

^b^N/A: not applicable.

^c^ISDN: integrated services digital network.

^d^RCT: randomized control trial.

^e^UK: United Kingdom.

^f^ATLS: advanced trauma life support.

^g^A&E: accident and emergency.

^h^OPD: outpatient department.

^i^ENT: ear, nose, and throat.

^j^NHS: National Health Service.

^k^DGH: district general hospital.

^l^DNA: did not attend.
